# HLAscan: genotyping of the HLA region using next-generation sequencing data

**DOI:** 10.1186/s12859-017-1671-3

**Published:** 2017-05-12

**Authors:** Sojeong Ka, Sunho Lee, Jonghee Hong, Yangrae Cho, Joohon Sung, Han-Na Kim, Hyung-Lae Kim, Jongsun Jung

**Affiliations:** 1 R&D center, Syntekabio, Inc., 5 Hwarang-ro 14-gil, Seongbuk-gu, Seoul 02792 South Korea; 2 Main office, Syntekabio, Inc., 187 Techno 2-ro, Yuseong-gu, Daejeon, 34025 South Korea; 3 Complex Disease and Genome Epidemiology Branch, Department of Epidemiology, School of Public Health, Seoul National University, Seoul, 08826 South Korea; 4 Department of Biochemistry, School of Medicine, Ewha Womans University, Seoul, 07985 South Korea

**Keywords:** HLA typing, Next-generation sequencing, Phasing issue, HLAscan

## Abstract

**Background:**

Several recent studies showed that next-generation sequencing (NGS)-based human leukocyte antigen (HLA) typing is a feasible and promising technique for variant calling of highly polymorphic regions. To date, however, no method with sufficient read depth has completely solved the allele phasing issue. In this study, we developed a new method (HLAscan) for HLA genotyping using NGS data.

**Results:**

HLAscan performs alignment of reads to HLA sequences from the international ImMunoGeneTics project/human leukocyte antigen (IMGT/HLA) database. The distribution of aligned reads was used to calculate a score function to determine correctly phased alleles by progressively removing false-positive alleles. Comparative HLA typing tests using public datasets from the 1000 Genomes Project and the International HapMap Project demonstrated that HLAscan could perform HLA typing more accurately than previously reported NGS-based methods such as HLAreporter and PHLAT. In addition, the results of *HLA-A*, −*B*, and *-DRB1* typing by HLAscan using data generated by NextGen were identical to those obtained using a Sanger sequencing–based method. We also applied HLAscan to a family dataset with various coverage depths generated on the Illumina HiSeq X-TEN platform. HLAscan identified allele types of *HLA-A*, −*B*, −*C*, −*DQB1*, and *-DRB1* with 100% accuracy for sequences at ≥ 90× depth, and the overall accuracy was 96.9%.

**Conclusions:**

HLAscan, an alignment-based program that takes read distribution into account to determine true allele types, outperformed previously developed HLA typing tools. Therefore, HLAscan can be reliably applied for determination of HLA type across the whole-genome, exome, and target sequences.

**Electronic supplementary material:**

The online version of this article (doi:10.1186/s12859-017-1671-3) contains supplementary material, which is available to authorized users.

## Background

The major histocompatibility complex (MHC) proteins play critical roles in regulating the adaptive immune system in vertebrates. Specifically, the MHC proteins participate in suppression and removal of pathogens by binding to foreign self-peptides and presenting antigens to receptors on other immune cells [1, 2]. Human MHC proteins are encoded by the human leukocyte antigen (*HLA*) locus, which maps to a 3.6 Mbp stretch on human chromosome 6p21.3. The *HLA* locus is one of the most complex regions of the human genome: although it constitutes only 0.3% of the genome, it makes up 1.5% of genes in OMIM, and 6.4% of genome-wide significant SNPs are located in this region [3]. Multiple genome-wide association studies have identified statistically significant associations between SNPs within *HLA* genes and disease phenotypes [3, 4], and shown that this region is associated with more diseases (mainly autoimmune and infectious) than any other region of the genome [1, 5]. In the clinic, acceptance or rejection of the graft after tissue transplantation is primarily determined by compatibility of *HLA* gene sequences between donor and recipient. Therefore, precise HLA typing is of great clinical importance, and a great deal of research effort has been devoted to the identification of HLA subtypes and development of typing methods [6–8]. Nonetheless, precise HLA typing remains very challenging due to the high degree of polymorphism among HLA genes [7], sequence similarity among these genes, and extreme linkage disequilibrium of the locus [9]. For example, according to the ImMunoGeneTics project (IMGT)/HLA database, over 3000 allele variants have been reported in the MHC class I *HLA-B* gene [7], and the alleles of *HLA-A*, *B*, and *C* exhibit high similarities.

For clinical purposes, HLA typing at the amino-acid level (four-digit) is necessary, because amino-acid differences among HLA proteins with the same antigenic peptide (two-digit) can lead to allogeneic responses. Established methods for HLA typing at this high resolution include polymerase chain reaction (PCR) using sequence-specific oligonucleotide (SSO) or Sanger sequencing–based typing (SBT). Although useful in routine clinical practice, these methods are low-throughput, labor-intensive, and expensive [8, 10]. As an alternative, targeted amplicon sequencing (also known as the PCR-NGS approach) was recently developed. This technology uses standard PCR to capture regions of interest, and the resultant amplicons are then subjected to next-generation sequencing (NGS). The method is relatively high-throughput and inexpensive compared with PCR-SSO and PCR-SBT, and enables highly accurate HLA typing by producing hundreds of base pairs of long sequence reads at high coverage depth [11–13]. Furthermore, over the past few years, genome-wide sequencing data such as whole-genome sequence (WGS) or whole-exome sequence (WES) became widely available as a result of various genome sequencing projects, e.g., the 1000 Genomes Project [14], NHLBI GO Exome Sequencing Project (https://esp.gs.washington.edu/), and UK10K project (http://www.uk10k.org/). Although most of the recently generated genome-wide datasets consist of short sequence reads (~101 bp), for reasons related to efficiency and cost, HLA typing from WGS or WES datasets is a feasible and efficient strategy for achieving accurate typing with existing resources [6, 15].

Several groups have developed methods for HLA typing using short sequence reads as input, and their approaches can be classified into two groups: the assembly approach, in which short reads are assembled into longer contigs, and the alignment approach, in which short reads are aligned to known reference allele sequences. Both methods have an elevated risk of detecting false-positive alleles resulting from phase ambiguity. In addition, the former method is time-consuming because it requires complex computational procedures. Despite these difficulties, advances in NGS have been accompanied by the development of multiple software packages capable of performing HLA typing using short reads, e.g., the assembly approach has introduced software such as HLAminer [16], HLAreporter [17], and ATHLATES [18], whereas the alignment approach has yielded programs such as PHLAT [15] and Omixon Target HLA [19]. Although recently published programs such as HLAreporter and PHLAT are able to predict HLA types quite accurately, their precision could still be improved. In this study, we developed an enhanced method, HLAscan, and compared its HLA typing performance with those of HLAreporter and PHLAT using multiple NGS datasets that were either publically available or newly generated in this study.

## Methods

### WES data from public genome datasets

Public WES datasets were utilized to verify HLAscan performance: specifically, FASTQ data for 10 samples from the 1000 Genomes Project (http://www.internationalgenome.org/) and 51 samples from the International HapMap Project (ftp://ftp.ncbi.nlm.nih.gov/hapmap/) were downloaded from the respective websites. For the 10 samples from the 1000 Genomes Project, HLA types were determined by a Sanger sequencing–based method reported elsewhere [18]. These data were used to evaluate the accuracy of the typing results generated by PHLAT and HLAreporter [15, 17]. Verified HLA types for the 51 HapMap samples were also reported previously [12, 20]. Previously, the HLAreporter algorithm was evaluated using HapMap data (18, 18, 11, 45, and 46 cases for *HLA-A*, *HLA-B*, *HLA-C*, *HLA-DRB1*, and *HLA-DQB1*, respectively) [17]. Analysis using these samples enabled comparison of the performance of HLAscan with typing results obtained by other methods. To avoid biasing the analysis in a manner that would have favored HLAscan, typing accuracy was evaluated using the values suggested in the original publications describing HLAreporter and PHLAT.

### Sequencing-based genotyping of *HLA-A*, −*B*, and *-DRB1*

Genomic DNA of five Korean subjects was extracted from white blood cells using the Blood DNA Extraction kit (Qiagen, Palo Alto, CA, USA). PCR-SBT was performed on *HLA-A*, −*B* (exons 2–4), and *-DRB1* (exon 2) using the SeCore A, B and DRB1 Locus Sequencing Kit (Invitrogen, Brown Deer, WI, USA). Data analysis was performed using the uTYPE HLA SBT software v3.0 (Invitrogen) and Sequencher (Gene Codes Corp., Ann Arbor, MI, USA). Detailed information on the subjects and the SBT-based HLA typing method were reported previously [21].

### NGS-based sequencing of HLA genes in samples from Korean subjects

To generate targeted sequencing data, all samples of total DNA were extracted from white blood cells using the Blood DNA Extraction kit. Five samples were sequenced using the NextGen sequencing system (MGH, Boston, MA, USA). For family data, nine families consisting of a total of 52 individuals participated in this study. Four families included two generations, including both parents and one or two offspring (three quads and one trio), and were sequenced at approximately 30× read depth. The other five families included three generations, and the members of each family were sequenced at three different coverage depths: 30×, 60×, and 90×. Genome sequence was determined using the HiSeq X-TEN system with the TruSeq DNA PCR-free library (Illumina, San Diego, CA, USA). Genomic DNA (500 μg) was sheared into 150–200 bp fragments on a Covaris sonicator (Covaris, Woburn, MA, USA), which generates dsDNA fragments with 3’ or 5’ overhangs. Following AMPureXP purification using magnetic beads (Beckman Coulter, Boulevard Brea, CA, USA), the double-stranded DNA fragments with overhangs were repaired using exonuclease and polymerase mix, and clones of appropriate sizes were selected using various ratios of sample purification beads in the AMPureXP system. Multiple indexing adaptors were ligated to the ends of the DNA fragments to prepare them for hybridization onto a flow cell. Prior to sequencing, the enriched DNA library with adaptor-modified ends was further amplified by PCR (six cycles, Herculase II fusion DNA polymerase) with pre-capture reverse PCR primers. The targeted genes were captured by hybridization of the amplified library with capture probes for 24 hrs at 65 °C. The hybridization mix was washed in the presence of magnetic beads (Streptavidin T1, Life Technologies). The eluted fraction was PCR amplified (16 cycles), and 30 index-tagged libraries were combined. The final library was sequenced on an Illumina HiSeq X-TEN platform with a paired-end run of 2 × 151 bp. The quality of each read was initially verified using the software embedded in the HiSeq X-TEN sequencer. A FASTQ file was generated for each tester sample for sequence alignment and converted to a BAM file for further analysis. (All FASTQ files are available on request.)

### Preprocessing for HLAscan: Alignment of sequence reads to HLA genes

HLAscan starts with sequence reads in FASTQ format for mapping to IMGT/HLA data. For targeted sequencing data, sequence reads can be used as direct input for HLAscan, whereas for WGS and WES data, it is necessary to select reads for HLA genes prior to running HLAscan. In comparison with targeted sequencing data, alignment of whole-genome/exome data directly to the IMGT/HLA database may miss some HLA reads. Nonetheless, this algorithm was adopted because alignment of HLA reads to the IMGT/HLA database is advantageous in regard to both time and computational processing without loss of predictive accuracy. Initial alignment was performed using bwa-mem v0.7.10-r789 with default options [22]. BWA-MEM is an accurate standard tool for aligning next-generation sequencing data to a reference sequence. In addition, it is a fast alignment tool; therefore, in our application, which involved many allele sequences in IMGT/HLA, BWA-MEM was the best fit for HLAscan. Sequence reads in the BAM file were sorted by reference coordinates using the FixMateInformation function, followed by removal of duplicate reads using MarkDuplicates in the Picard software package (version 1.68) (http://picard.sourceforge.net). Subsequently, identification of indels and re-alignment around these features were performed with the RealignerTargetCreator and IndelRealigner tools, respectively, and base-pair quality scores were recalibrated with BaseRecalibrator and PrintReads using the GATK software (version 3.3.0) ([23], http://www.broadinstitute.org). Throughout this processes, sequence reads corresponding to the exonic regions of *HLA* genes were selected based on an initial alignment generated using GATK with a whole-genome reference (GRCh37.p13). This filtering step does not classify the sequence reads into specific HLA genes.

**Fig. 1:**
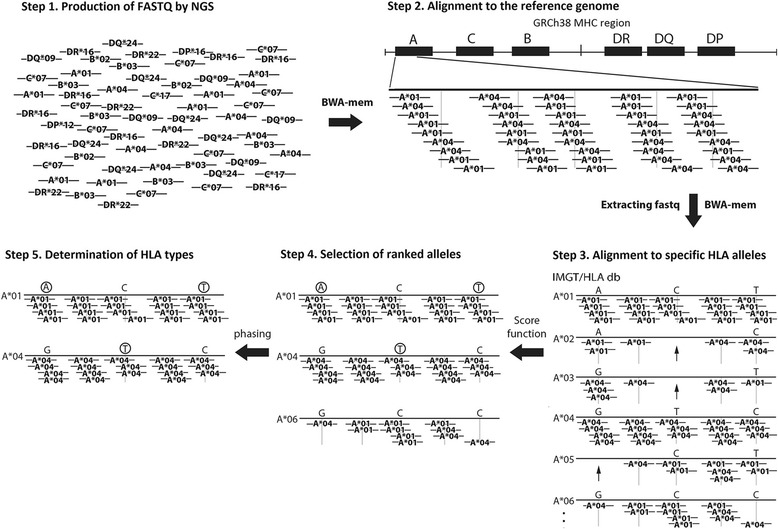
HLAscan workflow. The algorithm of HLAscan is explained schematically in five main steps. Step 1 depicts collection of read sequences of HLA genes produced from a sample. Step 2 demonstrates alignment of HLA-A gene read sequence to the human reference genome sequence. In step 3, HLA-A read sequences are aligned to specific allele types. From the candidate alleles, true allele types are determined by applying a score function (step 3 to step 4) and resolving phasing issues (step 4 to step 5). Gray vertical lines under reference sequences represent positions with sequence variance. Black arrows in alleles A*02, A*03, and A*05 of step 3 indicate genetic positions with no sequence reads aligned. Circled bases in step 4, A and T in A*01, and T in A*04 represent unique sequences that are not redundant with base sequences in any other ranked alleles

Analysis by HLAscan consisted of two steps. First, the selected reads were aligned with reference HLA alleles obtained from the IMGT/HLA database (http://www.ebi.ac.uk/ipd/imgt/hla/). This process extracted sequence reads exhibiting 100% identity with alleles in the database, and discarded the rest. Second, allele types were determined based on the numbers and distribution patterns of the reads on each reference target. A score function was optimized as described in the following section, and used to select candidate alleles prior to pinpointing correct alleles by resolving phasing issues (Fig. 1). Alignments were performed against exons 2, 3, 4, and 5 of class I HLA genes, and exons 2, 3, and 4 of class II genes. Typing was primarily performed with exons 2 and 3 for class I, and exon 2 for class II, HLA genes because, for many of the IMGT/HLA target alleles, sequence information is registered in the database only for these exons. When these exons did not provide enough specificity, the other exonic regions were taken into account for HLA inference. It takes nearly one hour for HLA typing of HLA-A, B, C, DR, and DQ when starting from BAM files of whole-genome and exome sequencing data, using a computer system (Intel Xeon CPU E5-2630 v2, 6 Cores).

### Score function for selecting candidate alleles by HLAscan

High polymorphism and the existence of numerous allele types for each gene make it difficult to handle the phasing issue, ultimately degrading the performance of HLAscan. Because the predictive accuracy of the HLAscan algorithm is higher when the number of candidate alleles is smaller, it is necessary to minimize the number of candidate alleles by eliminating as many false alleles as possible prior to handling the phasing issue. To filter false alleles out of the initial candidate allele group, HLAscan uses a score function that evaluates the distribution of aligned reads on the target region. ‘Read_*i*_’ was defined as the coordinate on a target sequence that matches the center of the *i-*th read when there are n reads (1 ≤ *i* ≤ n). ‘Read_*i*_’ can be calculated by [(start coordinate of *i-*th read + end coordinate of *i-*th read)/2] when a sequence is aligned from the position of the start coordinate of *i-*th read to the end coordinate of *i-*th read in the target sequence. The number of consecutive positions in the target sequence with no read_*i*_ is the distance between the centers of two adjacent reads, defined as D_*j*_ (1 ≤ *j* ≤ m).

Then, the score function is calculated as:

$\mathrm{Score}={\displaystyle \sum_{j=1}^{\mathrm{m}}{\left(\frac{\mathrm{D} j}{\mathrm{c}}\right)}^3}$, where c is a constant.

The constant can be defined based on the sequence depth and length of the reads. When sequencing depth in the target region was 30× with evenly distributed reads of 150 ntd, the distance between the centers of two adjacent reads would be 5 under ideal circumstances. With real NGS data (60× obtained by targeted sequencing or 30× obtained by WGS), the constant was typically set to 30 with the assumption that each position was covered an average of five times (5×). If the distance between the centers of two adjacent reads (D_*j*_) is longer than 30, D_*j*_/c will be higher than 1. Therefore, longer distance will reach to the penalty cutoff more easily by the third power of the distance. The exponent value was tested from 2 to 4, and it was found that the third power provided the best resolution between score function values. For this study, it was assumed that the average length of sequence reads was approximately 150 bp, and the constant c was set to 30. When an allele contains a 150 bp region (i.e., the length of one read) between the centers of two adjacent reads, D*j* would be 150 and the score function would be 125. HLAscan discarded alleles with scores above 125 for all analyses in this study. Examples of read alignment are shown in step 3 in Fig. 1. Alleles A*01 and A*04 are true alleles derived from actual sample DNA sequences, whereas the rest are false alleles generated from parts of true alleles. Considering the number of the aligned reads, and depth coverage, the score function in HLAscan evaluates whether aligned reads are distributed evenly, and among these candidates would select alleles A*01, A*04, and A*06. The other alleles were eliminated because positions without perfectly matching reads would have significantly increased their scores.

### Removal of duplicated alleles

The remaining alleles that passed the score function test were considered as candidate alleles. Although many false alleles would be eliminated by the score function, HLAscan further minimizes the number of candidates by defining duplicated alleles and removing them in the next step. Duplicated alleles can arise for two different reasons. First, when the sequence information of reads that map to two distinct alleles is perfectly identical, HLAscan groups these reads and generates a representative allele. All alleles that belong to this representative allele are then designated as duplicated alleles. Mapping of identical reads to different alleles occurs because some IMGT/HLA alleles possess exons that are indistinguishable from each other. For example, *HLA-A* alleles *02:01:01:01, *02:01:01:02 L, *02:01:01:03, and *02:01:01:04 share eight exons from exons 1 to 8. If *02:01:01:01 is the true allele, the other three alleles will have the same scores and pass the score function test. HLAscan virtually set allele *02:01:01 as a representative allele and discarded the four 8-digit alleles from the candidate list. Second, it is possible for all of the sequencing reads that map to one allele to constitute a subset of sequence reads that map to another allele. In this case, the former allele will be called a duplicated allele. Because the two alleles share high similarity, if one of them is the true allele, then the other would pass the score function test too. An additional algorithm was designed to select true alleles among these similar candidates, based on the assumption that true alleles are more likely to carry unique reads than false alleles. At this step, each candidate allele was evaluated to determine whether any sequence reads around the variant sequences were unique in the candidate. The unique sequence were counted, and candidates with unique sequence blocks were selected as candidate true alleles, whereas alleles without unique sequence blocks were discarded.

### Handling phase issues by HLAscan

Removal of duplicated alleles usually leaves several or fewer candidate alleles. The number of unique sequence reads on each of the candidate alleles is counted again, because the number of unique sequences in the candidate alleles may be miscounted due to the presence of false alleles that were removed at the previous step. Then, the first and second candidate alleles are determined based on which has a higher unique read count. Eventually, the system yields a heterozygote call if the two final candidate alleles possess uniquely aligned reads, or a homozygote call if only one allele possesses unique aligned reads. An example is provided in step 4 of Figure 1. Alleles A*01, A*04, and A*06 represent alignment with good depth coverage and relatively even read distribution. Although allele A*06 has reads that are common to allele A*01 or A*04, allele A*01 and A*04 both possess their own unique reads. In this case, HLAscan will select alleles A*01 and A*04 as the final HLA types.

## Results

### Predictions of 11 samples from the 1000 Genomes Project

We evaluated the performance of HLAscan by comparing the HLA types predicted by this algorithm with published data [18] for 10 individuals whose genome sequences are publically available from the 1000 Genomes Project (http://www.internationalgenome.org/). The score function cutoff was set to 125, and a higher cutoff did not improve prediction accuracy. We also compared the HLA types predicted by HLAscan with those obtained from two other algorithms, PHLAT [15] and HLAreporter [17]. This analysis encompassed 100 alleles, representing two alleles for each of five genes from 10 individuals (2 alleles × 5 genes × 10 individuals). PHLAT predicted HLA types for 100 alleles with an accuracy of 97% at the two-digit level and 95% at the four-digit level (Table 1 and Additional file 1: Table S1). HLAreporter predicted gene types with 98% accuracy at the two-digit level, but did not completely resolve phasing issues for 13 alleles; consequently, the software predicted multiple alleles including the correct one in each of these cases (Additional file 1: Table S1). HLAscan correctly predicted HLA alleles with 100% accuracy at both the two- and four-digit levels without ambiguity.

**Table 1:** Comparison of the performance of three methods using 1000 Genomes Project data

Methods	No. of examined alleles	Phase*	Wrong (2-digit)	Wrong (4-digit)	Accuracy (2-digit)	Accuracy (4-digit)
HLAreporter^1^	110	13	2	2	98%	98%
PHLAT^2^	100	-	3	5	97%	95%
HLAscan^3^	110	-	0	0	100%	100%

(^1^ Published [17]; ^2^ Published [15]; ^3^ In this study). * Multiple alleles were predicted due to ambiguous localization of sequence variants or unsolved phasing issues of various sequences

### Predictions of 51 HapMap samples

Next, we predicted HLA types for 51 individuals whose sequences were downloaded from the International HapMap Project (ftp://ftp.ncbi.nlm.nih.gov/hapmap/). Using previously published data as a reference for the correct typing results [12], we compared the results obtained with HLAscan with those generated by HLAreporter [17]. The score function cutoff was set to 125, and a higher cutoff did not improve prediction accuracy. Both HLAscan and HLAreporter predicted *HLA-A*, *HLA-B*, and *HLA-C* gene types with 100% accuracy at the two-digit level. At the four-digit level, HLAscan mistyped a HLA gene in two cases, whereas HLAreporter had accuracies of 80.5%, 83.3%, and 95.5% for *HLA-A*, *HLA-B*, and *HLA-C*, respectively (Table 2 and Additional file 2: Table S2). For class II genes, the differences in the results obtained by the two methods were marginal. The predictions of HLAscan agreed with the established results in 100% (two-digit) and 91.3% (four-digit) of cases for *HLA-DQB1*, and 96.7% (two-digit) and 95.6% (four-digit) for *HLA-DRB1* (Table 2). By comparison, HLAreporter had accuracies of 98.9% and 89.1% for *HLA-DQB1*, and 97.8% and 95.6% for *HLA-DRB1*.

**Table 2:** Comparison of HLA typing accuracies using HapMap data

Gene	A		B		C		DQB1		DRB1	
# alleles	36		36		22		92		90	
Methods	HLA reporter	HLA scan	HLA reporter	HLA scan	HLA reporter	HLA scan	HLA reporter	HLA scan	HLA reporter	HLA scan
Phase	5	-	6	-	4	-	0	-	2	-
Inaccurate (2-digit)	0	0	0	0	0	0	1	0	2	3
Inaccurate* (4-digit)	7	0	6	0	1	2	10	8	4	4
Accuracy (2-digit)	100%	100%	100%	100%	100%	100%	98.9%	100%	97.8%	96.7%
Accuracy (4-digit)	80.5%	100%	83.3%	100%	95.5%	90.9%	89.1%	91.3%	95.6%	95.6%

Comparison of typing results obtained using HLAreporter and HLAscan for *HLA-A*, −*B*, and *-C* (class I) and *HLA-DRB1* and *-DQB1* (class II). Verified HLA typing results were reported elsewhere [12]. * Inaccurate typing includes both mistyped and ambiguous cases

**Fig. 2:**
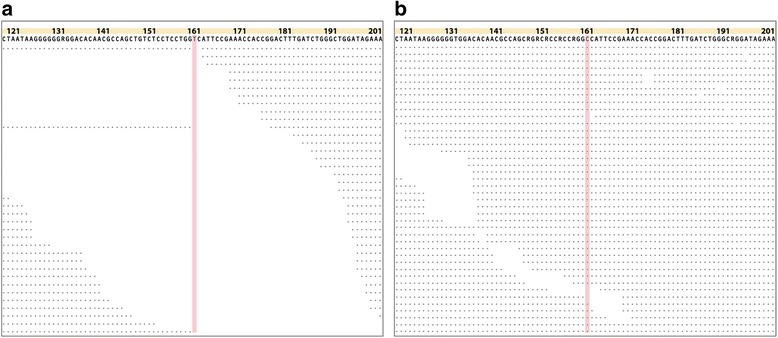
An example of mistyping DQB1*02:02:01:01 as DQB1*02:01:01:01. Sequence view showing actual alignment of sequence reads at exon 3 of DQB1*02:02:01:01 **a** and DQB1*02:02:01:01 (**b**). Consecutive dots under base calls represent sequence reads, and spaces without dots indicate that no sequence reads are aligned to the corresponding sequences. Pink spaces at position 161 show the status of sequence alignment over the SNP position that differs between DQB1*02:02:01:01 and DQB1*02:01:01:01. Actual mapping view of the sequence reads from NA11830 sample was generated in SAMtools tview

Further analysis of 12 cases of mistyping relative to the established results for HLA class II typing identified a particular subset of alleles: DQB1*02:01 (DQB1*02:02 in HLAscan) in six cases, DQB1*06:05 (DQB1*06:09 in HLAscan) in two cases, DRB1*15:01 (16:01 in HLAscan) in three cases, and DRB1*14:01 (DRB1*14:10 in HLAscan) in one case (Table 3). To understand the basis for the difference between the results, we scrutinized the actual alignments of sequence reads to the HLA genes, and found that HLAscan reported allele types with more uniform depth coverage throughout all sequence positions. For instance, DRB1*02:01:01:01 and DRB1*02:02:01:01 exhibit only one sequence difference at position 161 of exome 3 (Fig. 2). Many sequence reads supported ‘C’ at this position, whereas none supported ‘T’, disrupting the uniform distribution of the sequence reads. HLAscan predicted that DRB1*02:02:01:01 with uniform read distribution was correct. This type of read distribution difference explained 11 out of the 12 cases; the exception was DRB1*14:01. Thus, HLAscan precisely recognized even a one-base difference between HLA alleles and exhibited improved HLA typing accuracy in these datasets.

**Table 3:** Differences in typing results of HapMap data. Known HLA typing results were reported elsewhere [12]

Genes	Known HLA type		Predictions of HLAscan		# of the case
	Allele1	Allele2	Allele1 (correct)	Allele2 (mistyped)	
*DQB1*	xx:yy*	02:01	xx:yy*	*02:02*	6
	pp:qq*	06:05	pp:qq*	*06:09*	2
*DRB1*	15:01	15:01	15:01	*16:01*	3
	11:04	14:01	11:04	*14:10*	1

Asterisks (*) indicate alleles with multiple types

### Predictions of HLA allele types for five Korean subjects

For validation of HLAscan performance, we obtained samples from five Korean subjects whose HLA types were previously tested by SBT methods [21]. DNA samples were sequenced using the NextGen sequencing system at average coverage depth of 124× (Additional file 3: Table S3). HLAscan was performed to type *HLA-A*, *HLA-B*, and *HLA-DRB1*, and the results were compared with those generated by PCR-SBT. The results of HLAscan and PCR-SBT were perfectly concordant (Table 4), whereas HLAreporter mistyped four cases.

**Table 4:** Accuracy prediction of PCR-SBT, HLAreporter, and HLAscan using samples from five Korean subjects

Samples	Method	*HLA-A*		*HLA-B*		*HLA-DRB1*	
77072421 NS1512240004	PCR-SBT	02:06	02:10	40:02	55:02	04:05	11:01
	HLAreporter	02:10	02:10	40:02:01	55:02:01	04:05:01	11:01:01
	HLAscan	02:06:01	02:10	40:02:01	55:02:01	04:05:01	11:01:01
77072412 NS1512240008	PCR-SBT	24:02	31:01	35:01	51:02	09:01	09:01
	HLAreporter	24:82	31:01:02	35:42:02	51:02:02	09:01:02	09:01:02
	HLAscan	24:02:01	31:01:13	35:01:01	51:02:01	09:01:02	09:01:02
77072374 NS1512240012	PCR-SBT	02:01	33:03	15:01	44:03	09:01	13:02
	HLAreporter	02:01:01	33:03:01	15:01:01	44:03:11	09:01:02	13:02:01
	HLAscan	02:01:01	33:03:23	15:01:01	44:03:01	09:01:02	13:02:01
77072406 NS1512240016	PCR-SBT	11:01	26:01	44:02	46:01	09:01	13:01
	HLAreporter	11:01:01	26:01:01	44:02:01	46:01:01	09:01:02	13:01:01
	HLAscan	11:01:01:01	26:01:01:01	44:02:01	46:01:01	09:01:02	13:01:01
77072287 NS1512240020	PCR-SBT	02:01	02:06	13:01	40:02	08:02	12:02
	HLAreporter	02:01:01	02:01:01	13:01:01	40:02:01	08:02:01	12:02:01
	HLAscan	02:01:01	02:06:01	13:01:01	40:02:01	08:02:01	12:02:01

Typing results different from those obtained by SBT methods are marked in red

### Prediction of HLA types using family data with low sequence depth

Finally, to evaluate the utility of our software using data produced by widely used sequencing systems, we defined the HLA genotypes of nine families consisting of 52 individuals. Four families (#1, #2, #3, and #4), including three quartets and one trio, were sequenced at 30× read depth for all family members, whereas the other five families (#5, #6, #7, #8, and #9) were sequenced at three different coverage depths within each family (Additional file 7: Figures S1 and S2). This enabled us to test the effect of coverage depth on the accuracy of HLA typing by HLAscan. All samples were subjected to WGS on an Illumina HiSeq X-TEN sequencing system. Subsequent genotyping for *HLA-A*, −*B*, −*C*, −*DQB1*, and *-DRB1* was performed with HLAscan, generating the best results at the six-digit level under a functional score of 125 (Table 5 and Additional file 4: Table S4). Based on the typing results and family structure, we could infer the haplotype structure of HLA genes (Additional file 7: Figures S1 and S2). Families #5 and #6 included identical twins. Although the HLAscan algorithm can yield a final result of either two alleles (heterozygote) or one allele (homozygote), predictions of homozygote loci were sometimes inaccurate in light of the haplotype structure. Homozygosity without clear evidence of typing error was accepted. Ultimately, 504 (96.9%) out of 520 alleles were correctly identified, five (0.96%) alleles were non-identified, and 11 (2.1%) were mis-identified. Out of 52 individuals examined, samples from 10 individuals were sequenced at 90× depth, 17 at 60×, and 25 at 30×, with typing accuracies at the four-digit level of 100%, 96.5%, and 96%, respectively. The test of HLA typing at different average depths revealed that a certain level of depth may be necessary to minimize the typing error rate. For clinical use, utilization of sequencing data with good depth coverage, e.g., ≥ 90×, will be required.

**Table 5:** Accuracy of HLA typing using data from nine families. Results obtained at the four-digit level are summarized in this table. A total of 520 alleles were examined with 94% accuracy (489 correct), 2.3% (12 cases) missed, and 3.7% (19 cases) mistyped

9 families	90× (10 individuals)				60× (17 individuals)				30× (25 individuals)			
	# alleles	correct	missing	wrong	# alleles	correct	missing	wrong	# alleles	correct	missing	wrong
HLA-A	20	20	0	0	34	32	0	2	50	47	2	1
HLA-B	20	20	0	0	34	33	0	1	50	45	1	4
HLA-C	20	20	0	0	34	33	0	1	50	49	1	0
HLA-DQB1	20	20	0	0	34	33	0	1	50	50	0	0
HLA-DRB1	20	20	0	0	34	33	0	1	50	49	1	0
All	100	100	0	0	170	164	0	6	250	240	5	5
Percentage		100	0	0		96.5	0	3.5		96	2	2

### Relationship between read depth, score function, and HLAscan performance

Next, we created a receiver operating characteristic curve (ROC curve) to assess the accuracy of HLA typing as a function of depth coverage. For this purpose, we used a dataset consisting of 10 samples from the 1000 Genomes Project. For each sample, the *HLA-A*, *B*, *C*, *DRB1*, and *DQB1* genes were analyzed. The original file consisted of 50 cases (10 samples × 5 genes), including 49 cases with ≥ 100× coverage depth, of which 33 had ≥ 150× coverage.

**Fig. 3:**
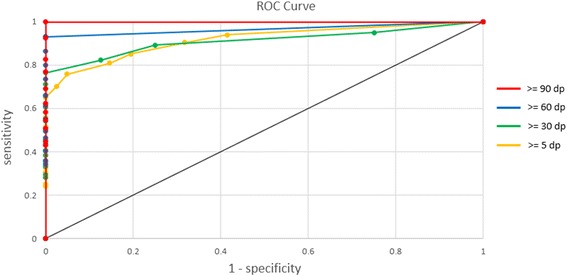
Analysis of typing accuracy as a function of coverage depth. ROC curve depicting sensitivity and specificity of HLA gene prediction by HLAscan depending on depth coverage. Sensitivity and (1-specificity) were calculated by the ROC Analysis software [24], and curves in different colors were plotted for accumulated datasets at different coverage depth cutoffs

To test the performance of HLAscan at various depths, we randomly selected 5%, 20%, 40%, 60%, 80% and 100% of all sequence reads in the original FASTQ file to test the performance of HLAscan at various depths for each gene and each sample. We then predicted the HLA types of the same individuals and calculated the specificity and sensitivity on data at each depth (Additional file 5: Table S5). The HLA prediction results at all depth coverages were combined and used to generate 4 new datasets, each of which were consisted of sequence reads over 5×, 30×, 60×, and 90× of coverage depth, respectively. For each dataset, sensitivity and specificity with regard to depth coverage changes were displayed by a ROC curve (Fig. 3). Our data indicated that the HLAscan algorithm provided sensitivity and specificity of 100% when the read depth was over 90× (red line in Fig. 3). The curve for reads with over 60× depth coverage exhibited a pattern similar to those obtained at higher depth, but with slightly lower sensitivity (blue line in Fig. 3). HLA prediction with reads at over 30× or 5× depth coverage (green and yellow line in Fig. 3, respectively) showed even lower sensitivity and specificity.

Then we examined HLA prediction accuracy by HLAscan along with sensitivity and specificity at various score function cutoffs, from 10 to 1000, to provide a guideline for setting the score cutoff (Additional file 6: Table S6). For sequences with higher depths (over 60% selection), the HLA inferences were perfectly correct. At 20% of read selection, prediction accuracy, sensitivity and specificity were 94% at all of the score cutoffs except for the cutoff 10, and these values did not dramatically changed dependent on the score cutoffs. At the cutoff 10, 91% of accuracy and sensitivity were observed. Five percent of read selection exhibited approximately 60% of accuracy and sensitivity, and 85% of specificity at most of score cutoffs, but 16% of accuracy and sensitivity, and 100% specificity were observed at the cutoff 10. These findings demonstrated that data with high read depth may not undergo filtration by the score function, and that HLA inference could still be carried out effectively via subsequent steps (i.e., removal of duplicated alleles and handling of the phasing issue). When sequencing depth was lower, sensitivity and specificity were slightly altered by low score cutoffs, but this effect was marginal. Therefore, we concluded that the score cutoff can be fixed for most of dataset, but read depth coverage would be a more critical factor for successful HLA inference by HLAscan.

## Discussion

High-resolution HLA typing is of critical importance in many applications. In particular, variant calling in highly polymorphic HLA regions is difficult when using short sequence reads at low sequencing depth. HLAscan performs alignment of HLA gene sequences with the IMGT/HLA database and takes into account a read distribution–based score function; in addition, the novel feature for elimination of false-positive alleles caused by phasing ambiguity was key to phasing of the two alleles. Consideration of read distribution by adopting the score function increased the accuracy of HLA typing compared with results obtained with previously reported software. In addition, the phasing issue was significantly improved by predicting final alleles with uniquely aligned sequence reads and discarding those that had reads in common with other candidates (Table 1 and Table 2).

Several parameters can influence performance of HLAscan. The major factors are coverage depth and length of sequence reads. The length of sequence reads is certainly important because the constant c is determined based on both sequence depth and read length. However, read length is fixed depending on the instrument used for sequencing. Our setting of the score function is based on 150 bp sequence reads, which is applicable to most short read sequences. Accordingly, we investigated effect of depth coverage in greater detail as a parameter that should be taken into account. The ROC curve enabled us to address the impact of coverage depth on HLA typing accuracy. Calculating sensitivity and specificity of HLA prediction with 4 datasets of different coverage depths, HLAscan predictions were nearly perfect at over 60× depth coverage. For clinical use it is recommended to utilize datasets with coverage depth over 90× to ensure 100% predictive accuracy. In addition, we examined whether score function would affect on HLA inference. Our result demonstrated that HLA prediction was not sensitive to alteration of the score cutoff value although higher score cutoff produced slightly better results at low depth coverage (Additional file 6: Table S6). To obtain best prediction results, it was more effective to run HLAscan with dataset at good depth coverage than to adjust the score cutoff on dataset with low depth coverage.

## Conclusion

HLAscan is an alignment-based multi-step HLA typing method considering read distribution. In this study we demonstrated that this new method not only outperformed the established NGS-based methods but also may complement sequencing-based typing methods when dealing with high-depth (~90×) short sequence reads. World-wide efforts in development of NGS technology have dramatically increased the availability of WGS and WES data. Accordingly, along with many existing germ line and somatic variant calling algorithms, HLAscan could be generally applied for variant calling in highly polymorphic regions.

## Additional files

Additional file 1: Table S1. HLA types for 10 1000G samples. (XLSX 15 kb)

Additional file 2: Table S2. HLA types for 51 HapMap samples. (XLSX 31 kb)

Additional file 3: Table S3. Sequencing depth for five samples from Korean subjects. (XLSX 11 kb)

Additional file 4: Table S4. Typing results from family data. (XLSX 31 kb)

Additional file 5: Table S5. Prediction of HLA types and calculation of specificity and sensitivity at different depths in 10 samples from 1000G datasets. (XLSX 40 kb)

Additional file 6: Table S6. Prediction of HLA types and calculation of specificity and sensitivity at different score cutoffs in 10 samples from 1000G datasets. (XLSX 63 kb)

Additional file 7: Figures S1. and **S2.** (DOC 785 kb)

## Abbreviations

HLA: Human Leukocyte Antigen
IMGT/HLA: ImMunoGeneTics project/Human Leukocyte Antigen
MHC: Major Histocompatibility Complex
NGS: Next-Generation Sequencing
PCR: Polymerase Chain Reaction
SBT: Sanger sequencing–Based Typing
SSO: Sequence-Specific Oligonucleotide
WES: Whole-Exome Sequence
WGS: Whole-Genome Sequence
