# Supplementary material for: HLAscan: genotyping of the HLA region using next-generation sequencing data
Source: BMC Bioinformatics. 2017 May 12;18:258. doi: 10.1186/s12859-017-1671-3 (PMC5427585; doi:10.1186/s12859-017-1671-3)
Supplement: Supplementary file 7 — and S2. (DOC 785 kb) [file 12859_2017_1671_MOESM7_ESM.doc]

Supplemental figures

**
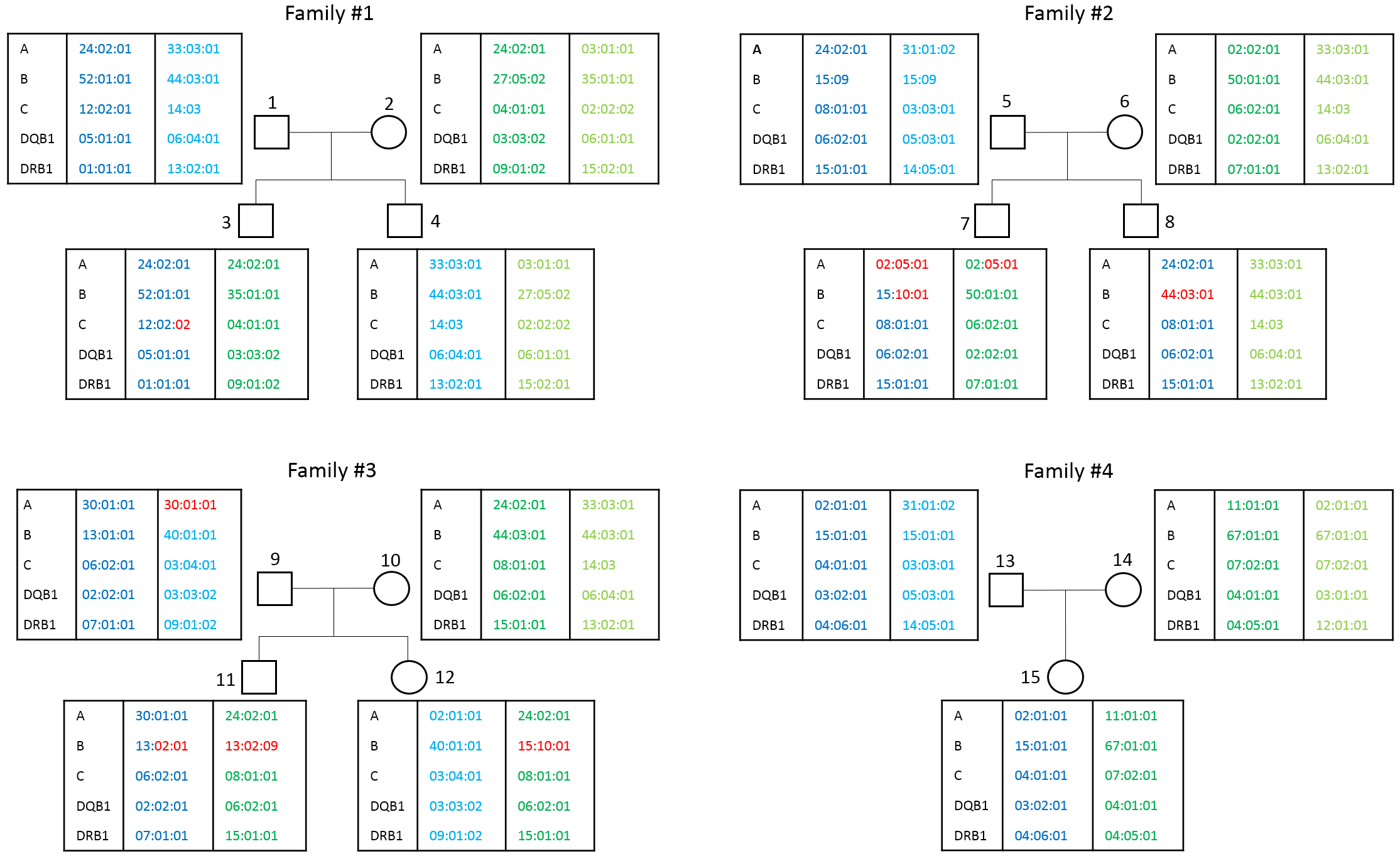
**

**Supplemental figure S1. HLA haplotypes in data from four families.** HLA genes were sequenced using the Illumina HiSeq X-TEN sequencing system. Haplotype structures of HLA loci were inferred from HLA gene typing results. Each haplotype is marked with a different color within a family. Red texts indicate mistyping.


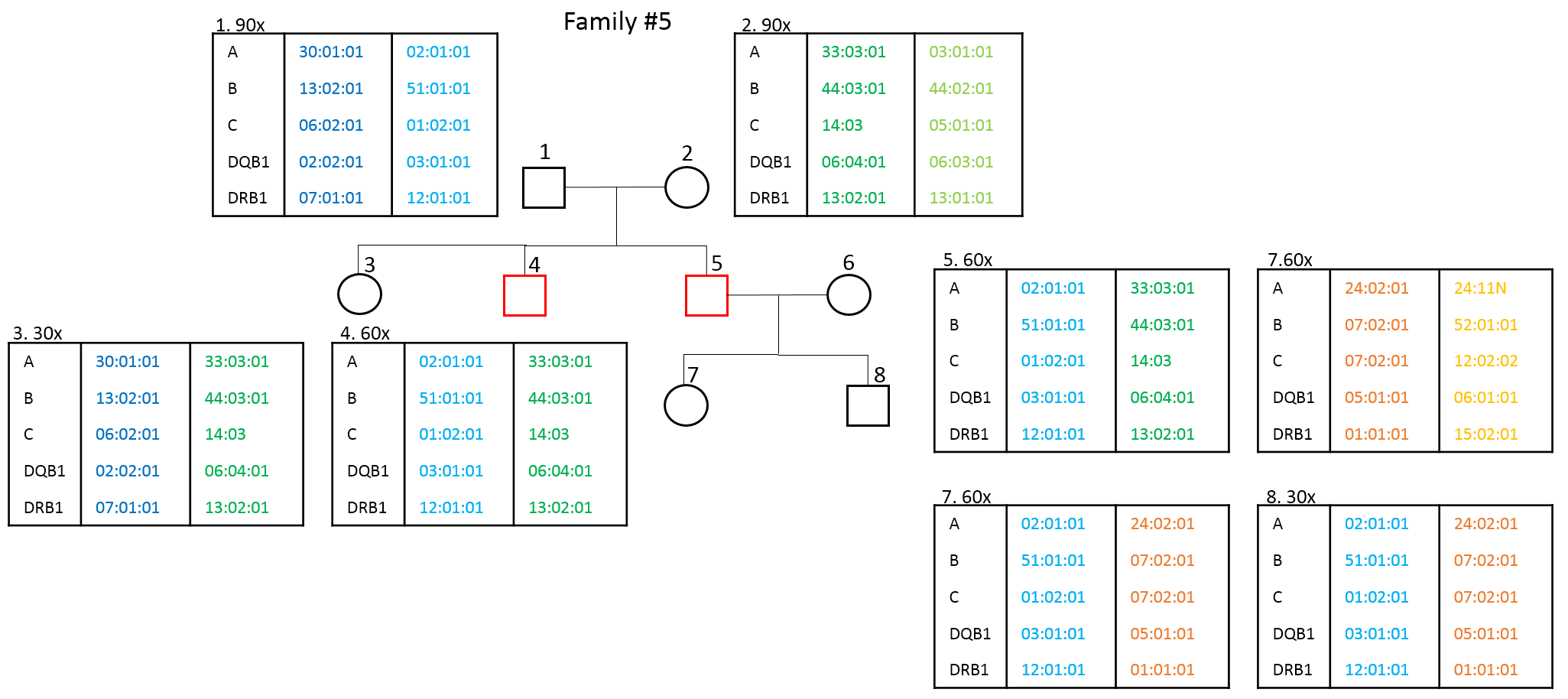


**
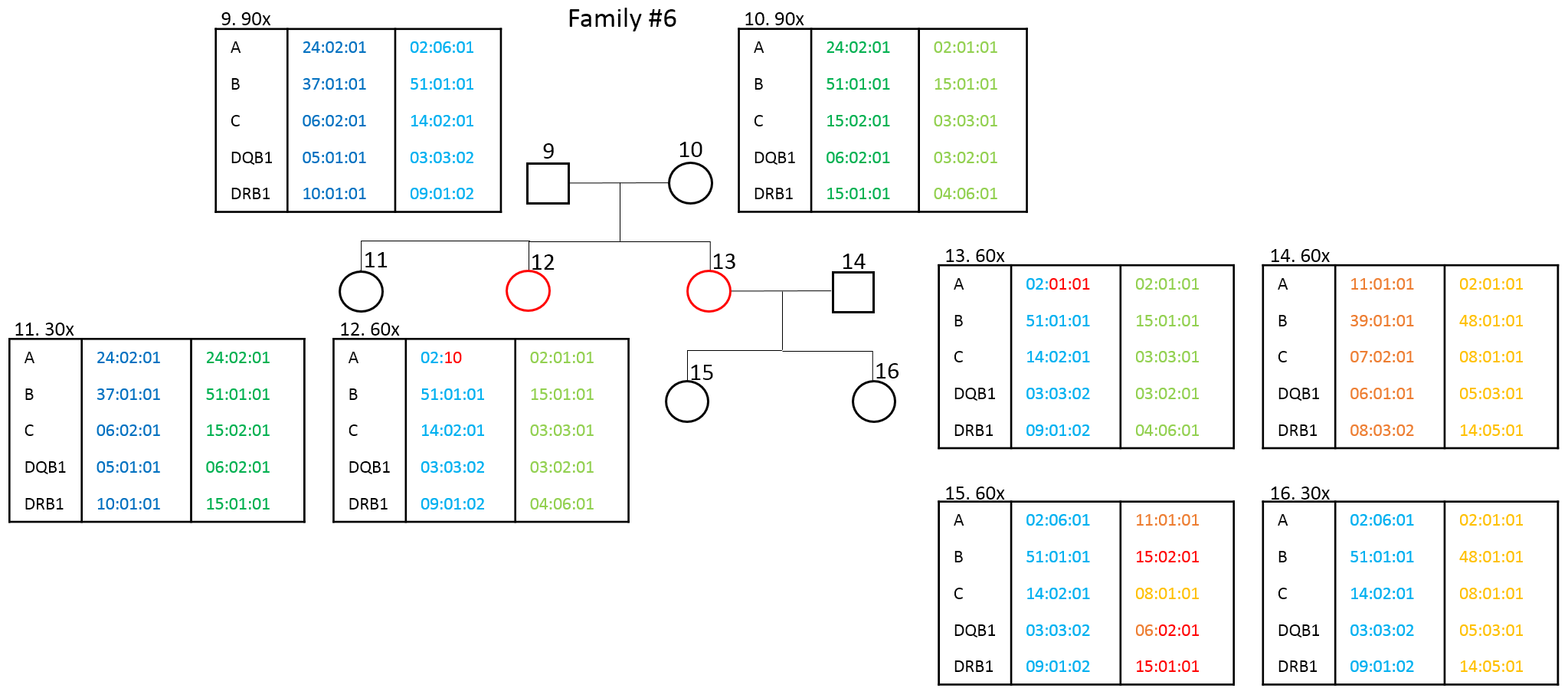
**

**
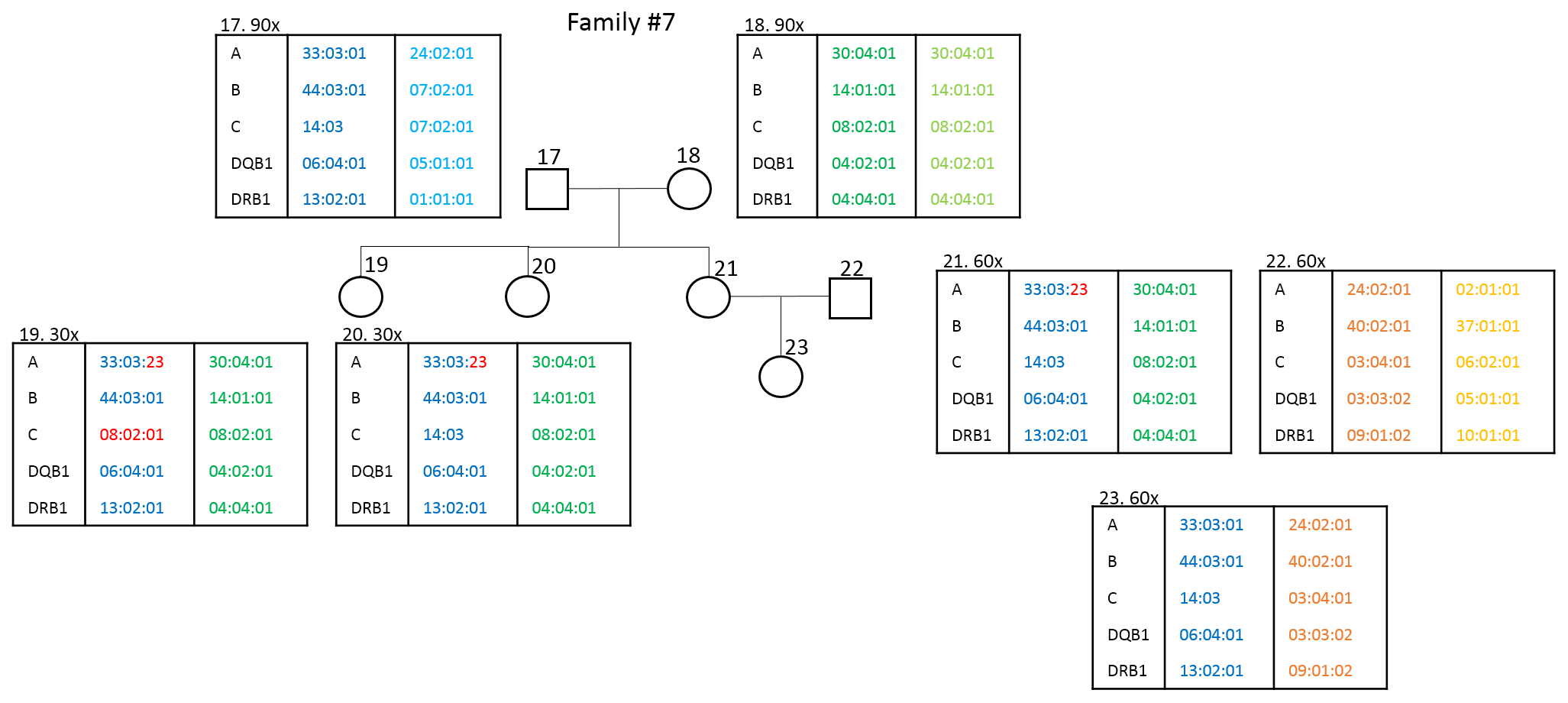
**

**
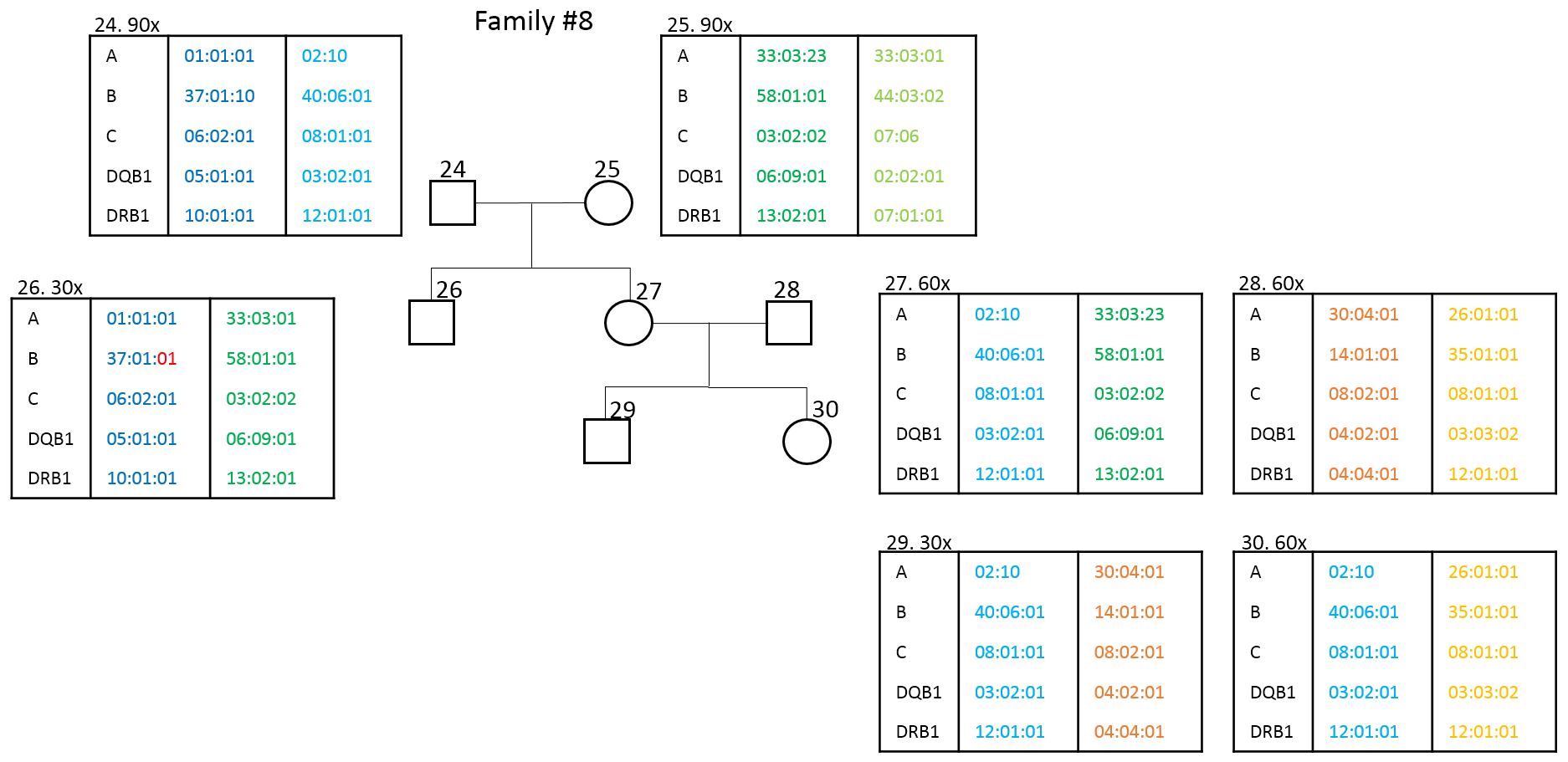
**

**
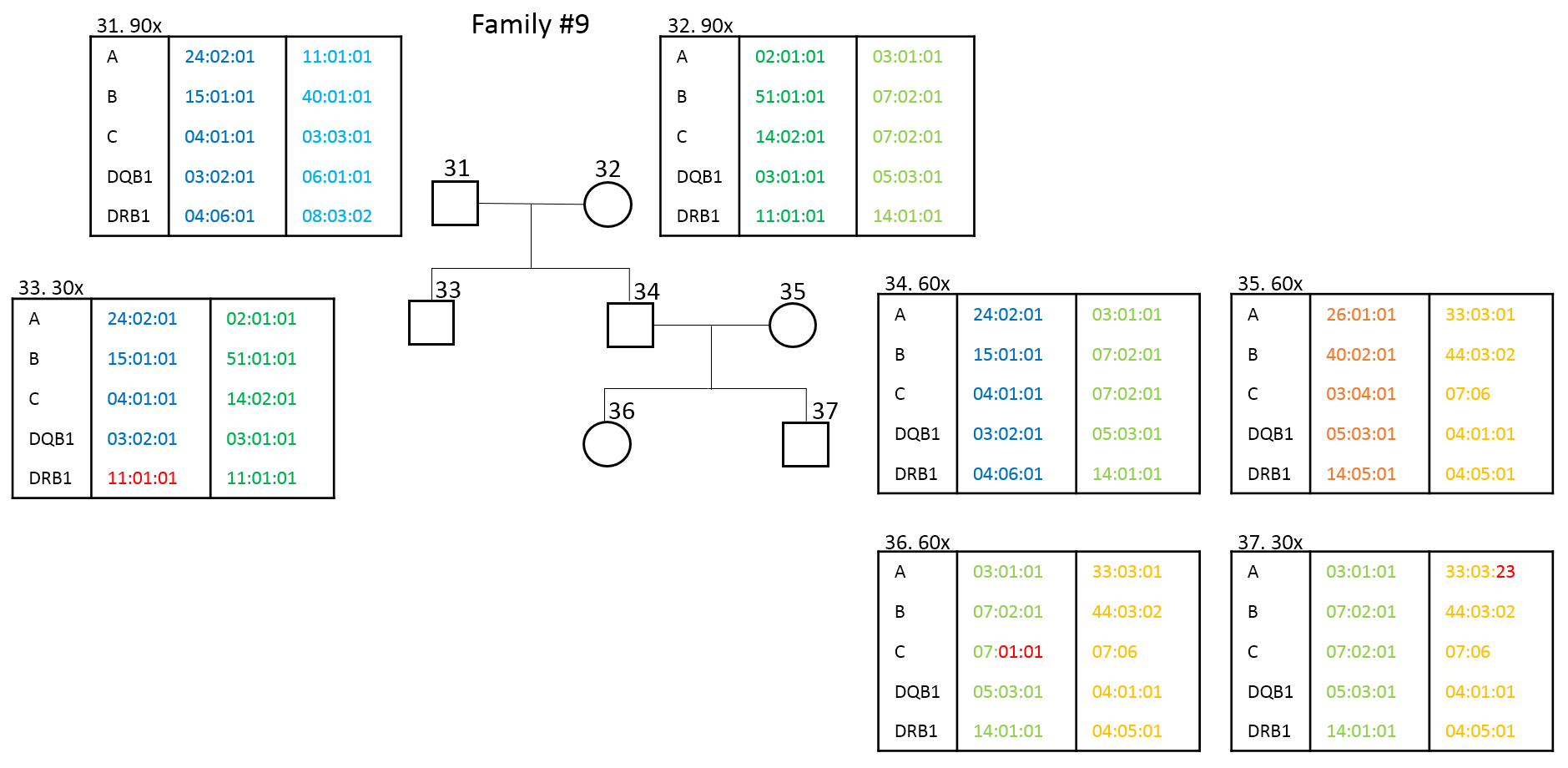
**

**Supplemental figure S2. The HLA haplotypes in five families with 3 generations.** HLA genes were sequenced using Illumina HiSeq X-TEN sequencing system. Haplotype structures of HLA loci were inferred by HLA gene typing results. Each haplotype was marked with different color within a family. Individual 4 and 5 in family #5, and 12 and 13 in family #6 (marked in red) are identical twins. HLA types in red texts are mistypings. Individual ID and the sequencing depth is denoted on top left of each table.
